# Dietary and Therapeutic Management of Glycogen Storage Disease Type IX: Analysis of a Systematic Review

**DOI:** 10.3390/children13050648

**Published:** 2026-05-05

**Authors:** Giulia Montanari, Andrea Zanaroli, Egidio Candela, Giacomo Biasucci, Federico Baronio, Rita Ortolano, Marcello Lanari

**Affiliations:** 1 Pediatric Unit, IRCCS Azienda Ospedaliero-Universitaria di Bologna, 40138 Bologna, Italy; giulia.montanari22@studio.unibo.it (G.M.); andrea.zanaroli@studio.unibo.it (A.Z.); federico.baronio@aosp.bo.it (F.B.); rita.ortolano@aosp.bo.it (R.O.); marcello.lanari@unibo.it (M.L.); 2 Specialty School of Pediatrics, Alma Mater Studiorum, University of Bologna, 40126 Bologna, Italy; 3 Department of Medical and Surgical Sciences, Alma Mater Studiorum, University of Bologna, via Massarenti, 11, 40126 Bologna, Italy; 4 Pediatrics and Neonatology Unit, Guglielmo da Saliceto Hospital, 29121 Piacenza, Italy; 5 Department of Medicine and Surgery, University of Parma, 43126 Parma, Italy

**Keywords:** glycogen storage disease type IX, GSD IX, GSD, metabolic disease, Glycosade, uncooked cornstarch, high-protein intake

## Abstract

**Highlights:**

**What are the main findings?**

- Dietary therapy plus uncooked cornstarch was the most commonly reported treatment for GSD IX, with reported improvement in hepatic outcomes and hypoglycemic episodes.
- Evidence on treatment strategies is limited and heterogeneous, based mainly on case reports and small case series, with scarce long-term follow-up.

**What are the implications of the main findings?**

- Management should be individualized, combining protein-enriched diets, cornstarch-based strategies, and monitoring for complications.
- Prospective multicentre studies and standardized protocols are needed to guide evidence-based care in GSD IX.

**Abstract:**

Background/Objectives: Glycogen storage disease type IX (GSD IX) is an inherited metabolic disorder characterized by marked clinical heterogeneity and variable severity. Dietary therapy is considered the cornerstone of management, but evidence on treatment strategies, efficacy, and safety remains limited. This study aimed to systematically synthesize available data on therapeutic approaches and clinical outcomes in GSD IX. Methods: A focused analysis of treatment-related data was conducted from a previously performed PRISMA-based systematic review. Clinical studies reporting treatment and follow-up data in genetically confirmed GSD IX patients were included. Results: Among 400 patients identified in the original review, 129 from 26 studies had treatment and follow-up data available. Dietary management combined with uncooked cornstarch (UCCS) was the most common approach (96.1%), with highly heterogeneous protocols. Hepatic manifestations improved in 59/129 (45.7%) of patients, and hypoglycemia in 45/129 (34.9%). Growth outcomes were variable, with catch-up growth in 14.0% and persistent impairment in 19.4%, although data were often missing. Muscle involvement was rarely assessed. No treatment-related adverse events were reported. However, disease-related complications were described, including liver cirrhosis, neurological involvement, osteopenia/osteoporosis, and two deaths in GSD IXa patients. Conclusions: Dietary therapy combined with UCCS remains the mainstay of treatment in GSD IX and is associated with improvement in key clinical domains. However, evidence is limited, heterogeneous, and largely based on small studies. Data on modified cornstarch formulations, such as Glycosade^®^, are scarce. Prospective studies and standardized treatment protocols are needed to support evidence-based management.

## 1. Introduction

Glycogen storage disease type IX (GSD IX, OMIM 306000) is an inherited disorder of glycogen metabolism characterized by impaired glycogen breakdown due to deficient activity of phosphorylase kinase (PhK), a key enzyme in glycogenolysis [1]. This disorder is characterized by a broad spectrum of clinical manifestations ranging from mild hepatic involvement to more severe phenotypes with hypoglycemia, growth impairment, and, in some cases, muscle symptoms. The underlying pathophysiology relates to the multimeric nature of PhK, composed of four subunits (*α*, *β*, *γ*, *δ*), encoded by distinct genes with tissue-specific isoforms [2]. Pathogenic variants in *PHKA2*, *PHKB*, and *PHKG2* underlie the hepatic subtypes IXa, IXb, and IXc, whereas variants in *PHKA1* cause the muscle-specific condition IXd [3,4]. While historically perceived as having a relatively benign course, it is now understood that GSD IX is associated with substantial long-term morbidity, including progressive liver fibrosis, cirrhosis [5,6,7], growth retardation, and, in some cases, neurological complications [8,9,10,11].

In a recent systematic review [12] that included 400 published cases of GSD IX, we provided a comprehensive overview of the clinical features and natural history of this disease, highlighting its marked phenotypic heterogeneity and the wide variability in disease severity. Hepatic forms are generally diagnosed in early childhood, usually before 5 years of age, whereas GSD IXd is typically diagnosed much later, with a mean age at diagnosis of 44.9 years, reflecting its milder, often underrecognized, muscular phenotype. Among the hepatic forms, IXc appears to be the most severe, with earlier and more pronounced hepatomegaly, more frequent hypoglycemia, and a greater potential for progressive liver involvement. By contrast, IXd is characterized predominantly by myalgia, cramps, exercise intolerance, and persistent elevation of creatine phosphokinase (CPK), with little or no hepatic or metabolic involvement [12]. These findings, together with the increasing number of diagnoses in recent years, have reshaped the clinical understanding of GSD IX and emphasized the importance of early recognition, molecular diagnosis, and long-term follow-up. However, unlike other inherited metabolic disorders, GSD IX is not currently included in most newborn screening programs and typically does not present with acute metabolic decompensation in the neonatal period [13]. Instead, it often manifests with a more insidious clinical course, which may delay diagnosis and contribute to under-recognition, particularly in milder forms.

Despite advances in next-generation sequencing (NGS) and improved phenotypic characterization, the therapeutic management of GSD IX remains insufficiently defined. Current therapeutic strategies are primarily supportive, aiming to maintain euglycemia and manage secondary metabolic disturbances. Dietary treatment is considered the cornerstone of care, with the main goals of preventing fasting intolerance and hypoglycemia, improving metabolic stability, supporting growth, and limiting long-term complications. In clinical practice, management usually relies on frequent meals, complex carbohydrates, alongside increased protein intake (approximately 2–3 g/kg/day or 20–25% of total caloric intake), distributed across meals and snacks throughout the day. In hepatic GSD IX, adequate protein intake may support endogenous gluconeogenesis, reduce reliance on fatty acid oxidation and ketone production, and contribute to growth and muscle metabolism [3]. Uncooked cornstarch (UCCS) is commonly used to help prevent catabolism and maintain stable blood glucose levels, while selected patients may require enteral feeding or extended-release cornstarch formulations such as Glycosade^®^, particularly when prolonged fasting tolerance or a reduction in overnight and daytime starch administrations is desired [3,14]. Although evidence in GSD IX remains limited, this approach has gained increasing clinical interest in hepatic glycogen storage diseases as a strategy to reduce treatment burden while maintaining metabolic stability [14].

The existing literature often lacks critical details on dosing, long-term efficacy, and patient-specific outcomes; therefore, significant gaps remain in our understanding of their optimal implementation. Evidence on treatment strategies for GSD IX remains limited, largely derived from case reports and small case series. Furthermore, the role of novel therapies and the management of unique subtypes, such as the muscle-specific GSD IXd, remain poorly defined. Given the evolving landscape of diagnosis—marked by the widespread adoption of NGS and an increasing number of identified cases—there is a pressing need to consolidate and critically appraise current treatment paradigms.

This follow-up review, built upon our previous systematic characterization of GSD IX [12], focuses specifically on therapeutic management. The present study aims to systematically review the available evidence on treatment strategies and reported clinical outcomes in patients with GSD IX, with particular attention to dietary interventions, cornstarch-based therapies, and their effects on hepatic manifestations, hypoglycemia, growth, and muscle involvement. By focusing on the therapeutic approach, this article highlights the need to complement diagnosis with more structured long-term therapeutic management and follow-up strategies.

## 2. Materials and Methods

### 2.1. Literature Search

This study represents a focused secondary analysis of treatment-related data extracted from a previously conducted systematic review on GSD IX. The original systematic review was performed in accordance with the Preferred Reporting Items for Systematic Reviews and Meta-Analyses (PRISMA) guidelines [15], using PubMed and Scopus databases. It was prospectively registered on PROSPERO (registration number: CRD42024622502). An overview is provided within Supplementary Table S1.

The original review included human clinical studies published up to 31 December 2024 and aimed to comprehensively describe the clinical, genetic, and diagnostic features of GSD IX. The full search strategy, selection process, and study characteristics have been previously reported [12].

All data analyzed in this study were extracted from previously published articles and are publicly available within the cited literature.

### 2.2. Study Selection

For the present study, only articles from the original dataset reporting therapeutic interventions and follow-up data were considered eligible for inclusion.

Eligible studies met the following criteria: a genetically confirmed diagnosis of GSD IX; description of at least one therapeutic intervention (e.g., dietary management, cornstarch therapy, enteral feeding, other); and availability of clinical or biochemical follow-up data.

**Figure 1:**
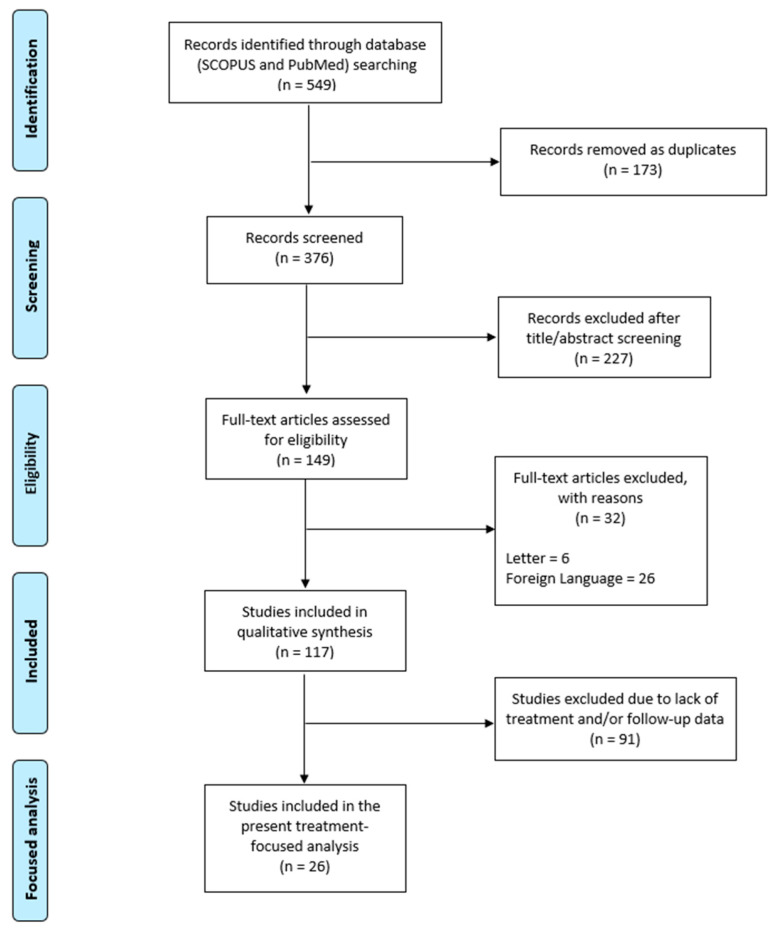
PRISMA flow diagram of the original systematic review and selection of studies included in the present treatment-focused analysis.

Studies lacking treatment details or outcome data were excluded from this focused analysis. Therefore, a modified PRISMA flow diagram—as reported in Figure 1—was constructed to illustrate the selection process for studies included in the present analysis.

### 2.3. Data Extraction and Quality Assessment

Data were extracted from the selected studies and systematically organized. The extracted variables included, when available, dietary strategies, feeding frequency, protein intake, use of UCCS or extended-release formulations (Glycosade^®^), and the need for enteral feeding. Clinical outcomes were categorized into hepatic manifestations, hypoglycemia recurrence, growth, and muscle involvement. In addition, reported adverse events were collected when available.

Due to the marked heterogeneity in study design, treatment protocols, outcome measures, and follow-up duration, a quantitative synthesis was not feasible. Therefore, a descriptive qualitative analysis was performed to summarize the reported therapeutic strategies and their associated clinical outcomes across studies. The analysis was based on aggregated data reported in the studies included. Because of the heterogeneous nature of the reports included, standardized outcome definitions were not consistently available. Outcomes were therefore grouped according to the descriptions provided in the original studies into hepatic, hypoglycemic, growth, muscle, and other metabolic or clinical domains. Hepatic improvement generally referred to reductions in hepatomegaly or improvement or even normalization in liver biochemistry; hypoglycemia improvement to reduction or resolution of reported episodes; the numerical threshold for hypoglycemia was often not specified, as the cut-off value differs across guidelines; growth improvement to reported catch-up growth, improved growth velocity, or improvement in standard deviation scores when available; muscle improvement referred to reported reduction in muscle weakness, exercise intolerance, myalgia; and lipid profile improvement mainly to reductions in triglycerides and/or total cholesterol. When precise thresholds or quantitative data were unavailable, qualitative author-reported outcomes were retained. No additional data beyond those reported in the original publications were generated or analyzed.

No formal assessment of reporting bias was conducted. The evidence included in this analysis is predominantly derived from case reports and small case series, characterized by heterogeneous reporting and variable completeness of clinical data. Similarly, the certainty of evidence for each outcome was not formally assessed (e.g., using GRADE), as the available data are descriptive and not suitable for structured comparative evaluation.

These limitations should be considered when interpreting the findings of the present study.

## 3. Results

A total of 26 studies reporting treatment and follow-up data in patients with GSD IX were included in the present analysis. These studies were published between 1998 and 2024, covering 26 years.

Among the 400 patients identified in the original systematic review, treatment and follow-up data were available for 129 individuals, who were therefore included in this focused analysis. Most studies consisted of case reports or small case series, with a predominance of hepatic subtypes. In particular, GSD IXa was the most represented subtype (88 patients, 68.2%), followed by GSD IXc (33 patients, 25.6%) and GSD IXb (8 patients, 6.2%). No patients with GSD IXd were included due to a lack of therapeutic and follow-up data.

A detailed analysis of treatment strategies and clinical outcomes is presented below in Table 1 [2,5,6,8,9,16,17,18,19,20,21,22,23,24,25,26,27,28,29,30,31,32,33,34,35,36], while aggregated findings are presented below (Table 2).

### 3.1. Therapeutic Strategies

Dietary management combined with UCCS represented the most commonly reported therapeutic approach, being used in 96.1% of cases across all three investigated subtypes, although dosing regimens were not standardized. High-protein diets and frequent feeding schedules were commonly adopted; however, treatment protocols varied considerably among studies, with reported protein intake ranging from 0.8 to 4 g/kg/day [19,25,26,30,31,33]. In a minority of cases, alternative approaches were described: four patients (two with GSD IXa and two with GSD IXb) were managed with dietary interventions alone [9,34], while one patient with GSD IXa received UCCS without a specifically defined dietary regimen [34].

### 3.2. Reported Outcomes

Improvement in hepatic manifestations was reported in 59 patients (45.7%), including reductions in hepatomegaly and transaminase levels. Conversely, hepatic outcomes remained unchanged or worsened in 28 patients (21.7%), particularly among those with GSD IXc (12 patients, 36.4% of this subtype), followed by GSD IXa (15 patients, 17.0%) and GSD IXb (1 patient, 12.5%). Follow-up data on hepatic outcomes were not reported for 42 of the 129 patients.

Similarly, reduction or resolution of reported hypoglycemic episodes was described in 45 patients (34.9%), whereas no improvement was observed in 16 patients (12.4%), including 10 patients with GSD IXa (11.4% of this subtype), 5 with GSD IXc (15.2%), and 1 with GSD IXb (12.5%). Data on hypoglycemic episodes at follow-up were not available for 68 patients (52.7%).

Growth outcomes were highly variable and frequently not reported (86/129 patients). Among those with available data, growth improvement, including catch-up growth or favorable auxological progression, was observed in 18 patients (14.0%), whereas persistent growth impairment or final short stature was reported in 25 patients (19.4%). This latter group included 17 patients with GSD IXa (19.3% of this subtype), 7 with GSD IXc (21.2%), and 1 with GSD IXb (12.5%).

Muscle involvement was rarely described, being reported in only two patients with GSD IXc, both presenting with mild muscle weakness that improved following treatment with a high-protein diet (3–4 g/kg/day) combined with uncooked cornstarch (UCCS, 0.5–1.8 g/kg/dose) [25]. However, in most cases, this outcome was not evaluated, reflecting the predominance of hepatic forms among the included subtypes.

An improvement in lipid profile (total cholesterol and triglycerides) was reported in 32 patients (24.8%), all treated with a combination of dietary management and UCCS.

Finally, in one patient with GSD IXa treated with combined dietary therapy and UCCS, proximal renal tubular acidosis was reported and appeared to improve at follow-up after treatment [9].

To provide a more structured overview of treatment effects across studies, outcomes were further summarized according to therapeutic approach (as illustrated in Table 2). This analysis highlights the proportion of patients showing improvement in key clinical domains: hepatic manifestations, hypoglycemia, and growth.

### 3.3. Adverse Events and Complications

Adverse events related to treatment were not reported in any of the included studies, regardless of the therapeutic approach (dietary management alone, UCCS alone, or combined therapy).

In contrast, several disease-related complications were described. Two patients with GSD IXa died, one due to aspiration and one due to sepsis [20]. Skeletal complications, including osteopenia or osteoporosis, were reported in four patients (one with GSD IXa, two with GSD IXb, and one with GSD IXc) [20]. Additional complications included progressive neurological disease in one patient with GSD IXa [9], seizures in one patient with GSD IXc [8], and liver cirrhosis in two patients with GSD IXa [5,6]. Overall, the reporting of complications was limited and heterogeneous across studies.

## 4. Discussion

This focused analysis of treatment-related data in GSD IX suggests an important role for dietary management in currently reported clinical practice and highlights the major limitations of the currently available evidence. While our previous systematic review primarily addressed the clinical characterization and natural history of GSD IX, the present manuscript specifically focuses on therapeutic strategies and reported clinical outcomes [12]. Dietary therapy, most often combined with UCCS, was the predominant approach across studies and was associated with reported improvement in hepatic manifestations and hypoglycemia in a substantial proportion of patients. These findings support its current use as the cornerstone of management in hepatic forms of GSD IX. However, our review of the existing literature suggests that the overall quality of evidence remains very low, as it is based almost exclusively on case reports and small case series. Treatment protocols, including protein intake and cornstarch regimens, were highly heterogeneous, and outcome definitions and follow-up assessments were inconsistent. The included studies span a 26-year period (1998–2024), during which therapeutic approaches appear to have evolved, yet without standardized protocols. This variability likely contributes to the heterogeneity observed in reported outcomes and limits the ability to identify optimal treatment strategies. An important finding of the present review is not only the heterogeneity of treatment approaches, but also the insufficient quality of treatment reporting across studies. Key elements such as protein prescription, cornstarch dose and adjustment, monitoring strategies, and objective outcome measures were frequently absent, limiting comparability and clinical interpretability.

Among subtypes, patients with GSD IXc appeared to have a relatively higher proportion of non-improved outcomes than those with GSD IXa and IXb, consistent with the more severe phenotype associated with PHKG2-related disease [17,37]. However, this observation should be interpreted with caution, given the small sample size and incomplete reporting. No patients with GSD IXd were included in this analysis, as this is a muscle-type glycogen storage disease; dietary management is unlikely to represent the main therapeutic strategy, and it likely reflects its milder, often underrecognized presentation, characterized mainly by subtle or exercise-related muscle symptoms rather than overt metabolic instability [38,39]. Nevertheless, the lack of treatment data in GSD IXd underscores an important gap in the literature and suggests that the therapeutic management of this form remains essentially unexplored.

Growth outcomes were particularly difficult to interpret. Follow-up data on growth were missing for most patients, and among those with available data, improvement was variable. This is not entirely unexpected, as growth in hepatic GSDs may improve spontaneously with age [40], making it difficult to distinguish treatment effect from the natural course of the disease. In addition, growth delay in GSD IX may reflect complex interactions among chronic fasting intolerance, ketosis, metabolic control, and age-related changes in glucose requirements [41,42]. For this reason, growth should probably be considered a clinically relevant but methodologically challenging outcome in future studies.

Notably, no treatment-related adverse events were reported. While this may suggest good tolerability and safety of dietary interventions, underreporting cannot be excluded. In contrast, several disease-related complications were described, including liver cirrhosis [5,6], neurological involvement [8,9], bone disease [20], and death in a small number of patients [20], underscoring the need for long-term monitoring.

### 4.1. Limitations

The findings of the present analysis should be interpreted in light of several limitations. First, the available evidence was derived almost exclusively from case reports and small case series, which carry inherent risks of publication bias and selective reporting. Second, treatment protocols were highly heterogeneous across studies, particularly regarding dietary composition, protein intake, UCCS dosing, monitoring strategies, and duration of follow-up, limiting direct comparability. There was also a lack of specific data on the dosage of therapeutic interventions.

Moreover, outcome definitions were inconsistent and frequently based on narrative clinical descriptions rather than standardized measures, especially for growth, hypoglycemia burden, and quality-of-life-related outcomes. Missing follow-up data were common for several clinically relevant domains, including hepatic progression, growth trajectories, and long-term complications.

Finally, as this was a secondary focused analysis based on previously published data [12], no individual patient-level re-evaluation or adjustment for potential confounders was possible. Accordingly, the present findings should be regarded as descriptive and hypothesis-generating rather than definitive evidence for specific treatment strategies.

### 4.2. Current Guidelines and Emerging Evidence

As stated above, limitations include the lack of comparative or prospective studies, the absence of standardized outcome measures, and the scarcity of data on alternative therapies, including extended-release waxy maize cornstarch formulations such as Glycosade^®^.

A more recent study [43], however, described improved glycemic profile in a patient with GSD IXa treated with Glycosade^®^, supporting growing interest in extended-release cornstarch also in this subtype.

Accordingly, the Glyde study [14], a prospective multicenter trial including patients with hepatic GSD (types I, III, VI, and IX), has provided a broader clinical framework for the use of Glycosade^®^ in hepatic glycogen storage diseases. In that study, extended-release cornstarch was associated with prolonged fasting tolerance, reduced daily starch administrations, and maintenance of stable metabolic control over long-term follow-up.

In that study, Glycosade^®^ was evaluated in 14 patients with GSD IX, was associated with a longer time to ketosis compared with UCCS (median 9.4 vs. 8.0 h, *p* = 0.005), and was preferred by 76% of participants for long-term use. Patients treated with Glycosade^®^ achieved stable metabolic control with fewer daily doses, in some cases avoiding daytime or school-time administration. In our analysis, no patients were treated with Glycosade^®^, highlighting a gap between the available case-based literature and emerging therapeutic strategies. Overall, the Glyde study suggests potential benefits of treatment burden and adherence without compromising metabolic outcomes. This aspect may be particularly relevant given the reported challenges in maintaining strict dietary regimens. Moreover, the Glyde study did not provide sufficiently granular data to define the efficacy of Glycosade^®^ across individual GSD IX subtypes, and no standardized dosing approach can currently be inferred for this specific disorder from the available literature. Therefore, while the available data suggest potential advantages in terms of fasting tolerance, adherence, and treatment burden, further subtype-specific prospective studies are needed before its role in GSD IX can be more clearly established.

Current international guidelines from the American College of Medical Genetics and Genomics (ACMG) [3] recommend dietary management, including frequent feeding, increased protein intake, and UCCS supplementation, as the mainstay of treatment for hepatic forms of GSD IX, despite the lack of standardized protocols and high-quality evidence. While the findings of the present analysis are consistent with these recommendations, several important gaps emerged. Protein intake (recommended 2–3 g/kg/day) and UCCS dosing were highly heterogeneous and often not reported in sufficient detail. Notably, a specific protein prescription was explicitly reported in only six of the included studies, highlighting the limited granularity of dietary reporting in the available literature. Among these, four described protein intakes broadly consistent with current ACMG recommendations (approximately 2–3 g/kg/day), whereas two earlier studies published in 2014 and 2017 reported higher intakes of 3–4 g/kg/day, without any difference in outcomes. Extended-release waxy maize cornstarch (Glycosade^®^), endorsed in the guidelines, was entirely absent from the included studies. Furthermore, while the ACMG guidelines emphasize home monitoring of blood glucose and ketones to guide treatment, systematic monitoring data were never reported. Finally, our findings support the guideline view that GSD IX is not a benign condition, with documented cases of cirrhosis, osteopenia, and mortality despite treatment.

### 4.3. Future Directions

Taken together, these findings point to several priorities for future research. It is also possible that the benefits of treatment are underestimated in the currently available literature. In expert clinical settings, timely and structured dietary management may yield greater improvement than suggested by published case-based data, which are often limited by incomplete reporting, variable follow-up, and non-standardized care pathways.

Multicenter prospective studies are needed to define more standardized dietary protocols and to assess treatment response using consistent outcome measures. Greater attention should also be paid to subtype-specific differences, long-term hepatic outcomes, growth trajectories, bone health, and patient-reported burden of therapy. The establishment of structured registries and collaborative networks could be especially valuable in a rare and heterogeneous disorder such as GSD IX, where individual centers are unlikely to accumulate sufficiently large cohorts on their own.

## 5. Conclusions

In conclusion, this treatment-focused analysis suggests that dietary management, most often combined with UCCS, remains the most reported therapeutic approach for patients with GSD IX. Available evidence indicates that this strategy may improve hepatic manifestations and hypoglycemia in a substantial proportion of patients, whereas growth outcomes appear more variable and are more difficult to interpret. However, the current literature is highly fragmented and is largely based on low-level evidence, with substantial heterogeneity in dietary regimens, outcome reporting, and follow-up.

No treatment-related adverse events were reported, but relevant disease-related complications were documented, supporting the need for careful long-term monitoring.

Overall, the available evidence is insufficient to support standardized treatment recommendations. Beyond summarizing currently reported approaches, this review highlights the urgent need for more standardized and detailed reporting of therapeutic management in GSD IX to improve the interpretability and applicability of future evidence.

The predominance of case reports and small case series, the marked heterogeneity of treatment protocols, and the frequent lack of follow-up data limited the strength of the conclusions. Therefore, the present findings should be interpreted primarily as descriptive and hypothesis-generating rather than definitive. Prospective studies, shared registries, and more structured reporting of therapeutic outcomes are needed to improve clinical management and to move toward a more evidence-based approach to treatment in GSD IX.

## Figures and Tables

**Table 1:** Summary of included studies, with treatment strategies, and clinical outcomes in patients with GSD IX. “N.R.” indicates data not reported in the original publication. Treatment details (including dietary composition, protein intake, UCCS dose, feeding schedule, and follow-up adjustments) are shown only when explicitly available from the source articles.

Article	N of Patients	Therapy (dose)	Improvement:					Complications
			Hepatic	Hypoglycemia	Growth/ Stature	Muscle	Other	
Fahiminiya et al. (2014) [16]	1 IXc	D + UCCS (N.R.)	Yes	N.R.	N.R.	N.R.	improved lipid profile	none
Waheed et al. (2020) [17]	10 IXc	D + UCCS (N.R.)	4 Yes 4 No 2 N.R.	7 Yes 3 N.R.	2 No 8 N.R.	10 N.R.	6 improved lipid profile	none
Shao et al. (2022) [18]	1 IXc	D + UCCS (N.R.)	No	Yes	Yes	N.R.	improved lipid profile	none
Karande et al. (2016) [19]	1 IXa	D (2.8 g/kg/day protein) + UCCS (1 g/kg/dose)	Yes	Yes	Yes	N.R.	improved lipid profile	none
İnci et al. (2022) [20]	14 IXa 3 IXb 6 IXc	D + UCCS (N.R.)	2 Yes (2 IXa) 2 No (1 IXa; 1 IXc) 19 N.R.	2 No (1 IXc; 1 IXa) 21 N.R.	23 N.R.	23 N.R.	23 N.R.	1 IXa died due to aspiration; 1 IXa died due to sepsis; 4 (1 IXa + 2 IXb + 1 IXc) osteopenia/osteoporosis
Li et al. (2021) [21]	2 IXa	D + UCCS (N.R.)	2 Yes	2 N.R.	2 N.R.	2 N.R.	2 N.R.	none
Mori et al. (2022) [22]	1 IXa	D + UCCS (N.R.)	Yes	N.R.	N.R.	N.R.	N.R.	none
Estrada (2020) [23]	1 IXa	D + UCCS (N.R.)	Yes	Yes	Yes	N.R.	improved lipid profile	none
Degrassi et al. (2021) [24]	8 IXa 1 IXb 3 IXc	D + UCCS (N.R.)	12 No	12 No	12 No	12 N.R.	12 N.R.	none
Burwinkel et al. (1998) [9]	3 IXa	D + UCCS (N.R.)	3 N.R.	1 Yes 2 N.R.	1 Yes 2 N.R.	3 N.R.	1 proximal renal tubular acidosis seemingly improved	1 progressive neurologic disease
	1 IXa	D	Yes	Yes	Yes	N.R.	N.R.	none
Burwinkel et al. (2003) [8]	3 IXc	D + UCCS (2 g/kg/day)	3 No	2 Yes 1 No	1 Yes 1 No 1 N.R.	3 N.R.	3 N.R.	1 seizures
Beyzaei et al. (2022) [5]	1 IXa	D + UCCS (1.5 g/kg/day)	No	N.R.	N.R.	N.R.	improved lipid profile	Liver cirrhosis
Bali et al. (2014) [25]	5 IXc	D (3–4 g/kg/day protein) + UCCS (0.5–1.8 g/kg/dose)	5 N.R.	5 Yes	2 Yes 3 N.R.	2 Yes 3 N.R.	5 N.R.	none
Kim et al. (2015) [2]	1 IXa	D + UCCS (N.R.)	No	Yes	N.R.	N.R.	N.R.	none
Tsilianidis et al. (2013) [26]	2 IXa	D (2.5 g/kg/day protein) + UCCS (3 times/day)	2 Yes	2 Yes	2 Yes	2 N.R.	2 improved lipid profile	none
Zhu et al. (2019) [27]	1 IXa	D + UCCS (N.R.)	Yes	Yes	N.R.	N.R.	N.R.	none
Fu et al. (2019) [28]	1 IXa	D + UCCS (N.R.)	N.R.	Yes	Yes	N.R.	N.R.	none
Johnson et al. (2012) [6]	1 IXa	D + UCCS (N.R.)	No	No	N.R.	N.R.	N.R.	Liver cirrhosis
Li et al. (2018) [29]	1 IXc	D + UCCS (N.R.)	Yes	Yes	N.R.	N.R.	N.R.	none
Beyzaei et al. (2021) [30]	1 IXb	D (2.5 g/kg/day protein) + UCCS (5 times/day)	Yes	Yes	N.R.	N.R.	N.R.	none
Zamanfar et al. (2024) [31]	1 IXb	D (>2 g/kg/day protein) + UCCS (N.R.)	Yes	N.R.	Yes	N.R.	N.R.	none
Vanduangden et al. (2024) [32]	1 IXa	D + UCCS (N.R.)	Yes	N.R.	No	N.R.	N.R.	none
Bali et al. (2017) [33]	12 IXa	D (3–4 g/kg/day protein) + UCCS (0.4–2 g/kg/dose)	12 Yes	12 N.R.	12 N.R.	12 N.R.	12 N.R.	none
Roscher et al. (2014) [34]	4 IXa 3 IXc	D + UCCS (0.8–2.4 g/kg)	1 Yes (IXc) 6 N.R.	3 Yes (2 IXa; 1 IXc) 4 N.R.	1 No (IXc) 6 N.R.	7 N.R.	7 N.R.	none
	1 IXa 2 IXb	D	1 Yes (IXa) 2 N.R.	1 Yes (IXb) 2 N.R.	3 N.R.	3 N.R.	3 N.R.	none
	1 IXa	UCCS (1.3 g/kg)	Yes	Yes	N.R.	N.R.	N.R.	none
Zhang et al. (2017) [35]	17 IXa	D + UCCS	12 Yes 1 No 4 N.R.	17 N.R.	17 N.R.	17 N.R.	5 improved lipid profile	none
Achouitar et al. (2011) [36]	14 IXa	D + UCCS (1–3 g/kg)	12 Yes 2 No	14 Yes	6 Yes 8 No	14 N.R.	14 improved lipid profile	none

D: Diet. UCCS: Uncooked corn starch. N.R.: Not reported. Yes = improved. No = unchanged/worsened.

**Table 2:** Treatment-specific summary of clinical outcomes in GSD IX.

Therapy	N Patients (%)	Hepatic Improvement	Hypoglycemia Improvement	Growth Improvement
Diet + UCCS	124 (96.1%)	56/124 (45.2%)	42/124 (33.9%)	17/124 (13.7%)
Diet alone	4 (3.1%)	2/4 (50%)	2/4 (50%)	1/4 (25%)
UCCS alone	1 (0.8%)	1/1 (100%)	1/1 (100%)	N.R. *
Total	129 (100%)	59/129 (45.7%)	45/129 (34.9%)	18/129 (14.0%)

Data are presented as absolute numbers; proportions should be interpreted cautiously due to the small number of patients in some subgroups. Outcome categories were based on author-reported definitions in the original studies and were not standardized across publications. * N.R.: Not reported.

## Data Availability

All clinical data and materials are available in our Pediatric Unit.

## Supplementary Materials

The following supporting information can be downloaded at: https://www.mdpi.com/article/10.3390/children13050648/s1; Table S1. PRISMA 2020 statement: an updated guideline for reporting systematic reviews.

## Abbreviations

The following abbreviations are used in this manuscript:

GSD	Glycogen storage disease
PhK	Phosphorylase kinase
CPK	Creatine phosphokinase
NGS	Next-generation sequencing
UCCS	Uncooked cornstarch
ACMG	American College of Medical Genetics and Genomics
