# Supplementary material for: Dietary and Therapeutic Management of Glycogen Storage Disease Type IX: Analysis of a Systematic Review
Source: Children (Basel). 2026 May 5;13(5):648. doi: 10.3390/children13050648 (PMC13205120; doi:10.3390/children13050648)
Supplement: Supplementary file 1 [file children-13-00648-s001.zip › children-4269622-supplementary.pdf]

Supplementary Table S1. PRISMA 2020 statement: an updated guideline for reporting systematic reviews.

Topic	No.	Item	Location where item is reported
**TITLE**			
**Title**	1	Identify the report as a systematic review.	Title
**INTRODUCTION**			
**Rationale**	3	Describe the rationale for the review in the context of existing knowledge.	Discussion
**Objectives**	4	Provide an explicit statement of the objective(s) or question(s) the review addresses.	Background
**METHODS**			
**Eligibility criteria**	5	Specify the inclusion and exclusion criteria for the review and how studies were grouped for the syntheses.	Study Selection
**Information sources**	6	Specify all databases, registers, websites, organisations, reference lists and other sources searched or consulted to identify studies. Specify the date when each source was last searched or consulted.	Study Design
**Search strategy**	7	Present the full search strategies for all databases, registers and websites, including any filters and limits used.	Study Design
**Selection process**	8	Specify the methods used to decide whether a study met the inclusion criteria of the review, including how many reviewers screened each record and each report retrieved, whether they worked independently, and if applicable, details of automation tools used in the process.	Study Selection
**Data collection process**	9	Specify the methods used to collect data from reports, including how many reviewers collected data from each report, whether they worked independently, any processes for obtaining or confirming data from study investigators, and if applicable, details of automation tools used in the process.	Data Extraction
**Data items**			
	10	List and define all other variables for which data were sought (e.g. participant and intervention characteristics, funding sources). Describe any assumptions made about any missing or unclear information.	Data Extraction

Supplementary Table S1. PRISMA 2020 statement: an updated guideline for reporting systematic reviews.

Topic	No.	Item	Location where item is reported
**Study risk of bias assessment**	11	Specify the methods used to assess risk of bias in the included studies, including details of the tool(s) used, how many reviewers assessed each study and whether they worked independently, and if applicable, details of automation tools used in the process.	Data Extraction
**Effect measures**	12	Specify for each outcome the effect measure(s) (e.g. risk ratio, mean difference) used in the synthesis or presentation of results.	Not applicable
**Synthesis methods**	13	Describe the processes used to decide which studies were eligible for each synthesis (e.g. tabulating the study intervention characteristics and comparing against the planned groups for each synthesis (item 5)).	Data Synthesis and Quality Assessment
**Reporting bias assessment**	14	Describe any methods used to assess risk of bias due to missing results in a synthesis (arising from reporting biases).	Study design
**Certainty assessment**	15	Describe any methods used to assess certainty (or confidence) in the body of evidence for an outcome.	Data Synthesis and Quality Assessment
**RESULTS**			
**Study selection**	16	Describe the results of the search and selection process, from the number of records identified in the search to the number of studies included in the review, ideally using a flow diagram.	Results AND Supplementary figure 1
**Study characteristics**	17	Cite each included study and present its characteristics.	Supplemental table 2
**Risk of bias in studies**	18	Present assessments of risk of bias for each included study.	Supplemental table 2
**Results of individual studies**	19	For all outcomes, present, for each study: (a) summary statistics for each group (where appropriate) and (b) an effect estimate and its precision (e.g. confidence/credible interval), ideally using structured tables or plots.	Supplemental table 2
**Results of syntheses**	20	For each synthesis, briefly summarise the characteristics and risk of bias among contributing studies.	Results
**Reporting biases**	21	Present assessments of risk of bias due to missing results (arising from reporting biases) for each synthesis assessed.	Not applicable
**Certainty of evidence**	22	Present assessments of certainty (or confidence) in the body of evidence for each outcome assessed.	Supplemental table 2

Supplementary Table S1. PRISMA 2020 statement: an updated guideline for reporting systematic reviews.

Topic	No.	Item	Location where item is reported
**DISCUSSION**			
**Discussion**	23	Provide a general interpretation of the results in the context of other evidence.	Discussion
**OTHER INFORMATION**			
**Registration and protocol**	24	Provide registration information for the review, including register name and registration number, or state that the review was not registered.	Statements. Statement of Ethics for systematic reviews
**Support**	25	Describe sources of financial or non-financial support for the review, and the role of the funders or sponsors in the review.	Statements. Funding Sources
**Competing interests**	26	Declare any competing interests of review authors.	Statements. Conflict of Interest Statement
**Availability of data, code and other materials**	27	Report which of the following are publicly available and where they can be found: template data collection forms; data extracted from included studies; data used for all analyses; analytic code; any other materials used in the review.	Statements. Data Availability Statements
